# Comparative Analysis between SPECT Myocardial Perfusion Imaging and CT Coronary Angiography for Diagnosis of Coronary Artery Disease

**DOI:** 10.1155/2012/253475

**Published:** 2012-07-16

**Authors:** Jian-ming Li, Ting Li, Rong-fang Shi, Li-ren Zhang

**Affiliations:** ^1^Department of Nuclear Medicine, Tianjin Medical University, Cardiovascular Clinical Institute and TEDA International Cardiovascular Hospital, Tianjin 300457, China; ^2^Department of Radiology, Tianjin Medical University, Cardiovascular Clinical Institute and TEDA International Cardiovascular Hospital, Tianjin 300457, China

## Abstract

The study aims to discuss the relationship and difference between myocardial perfusion imaging (MPI) using SPECT and CT coronary angiography (CTCA) for diagnosis of coronary artery disease (CAD). Five hundred and four cases undergoing MPI and CTCA were comparatively analyzed, including fifty six patients undergoing invasive coronary angiography in the same period. Among patients with negative MPI results, negative or positive CTCA occupied 84.7% or 15.3%, respectively. Among patients with positive MPI, positive or negative CTCA occupied 67.2% or 32.8%, respectively. Among patients with negative CTCA, negative or positive MPI occupied 94.4% or 5.6%, respectively. Among patients with positive CTCA, positive or negative MPI occupied 40.2% or 59.8%, respectively. Negative predictive value was relatively higher than the positive predictive value for positive CTCA eliminating or predicting abnormal haemodynamics. And there was no significant difference for sensitivity, specificity, and accuracy of MPI or CTCA in diagnosing CAD. Both MPI and CTCA have good diagnostic performance for CAD. They provide different and complementary information for diagnosis and evaluation of CAD, namely, detection of ischemia versus detection of atherosclerosis, which are quite different but have a definite internal link for each other.

## 1. Introduction

Myocardial perfusion imaging (MPI) with single-photon emission computer tomography (SPECT) is the most commonly used and well-documented noninvasive method for diagnosis and risk stratification of coronary artery disease (CAD) [1]. The location and extent of ischemia can be reliably and semiquantitatively obtained using MPI, which plays an important role for patient management. The latest advancements for computed tomography (CT), such as faster gantry, multidetector array, and even dual-source detectors, make it possible to noninvasively and intuitively obtain anatomic morphology of coronary arteries, especially contributing to identifying the magnitude, distribution, and composition of coronary atherosclerosis. It has been documented that CT coronary angiography (CTCA) has high accuracy in detection of obstructive CAD comparing with invasive coronary angiography (ICA) [2]. As noninvasive diagnostic methods of CAD for both MPI and CTCA, how to correctly recognize the respective role and relationship between them in diagnosing and evaluating CAD is a very important question concerned by clinicians. This study aims to discuss and evaluate the relationship and difference between SPECT MPI and CTCA through comparative analysis.

## 2. Materials and Methods

### 2.1. Study Population

Five hundred and four consecutive patients with suspected or known CAD were enrolled in this study. All cases underwent CTCA and MPI within 30 days. The mean age of the study population was 56.7 ± 8.7 y, with 326 patients being male (64.7%). Fifty six patients (man 56 women 18) among all cases also underwent ICA within 30 days, and the mean age for the group was 57.5 ± 9.2 y. ICA was finally performed after CTCA and MPI, with the sequence of CTCA and MPI depending on the individual clinical situation. The exclusion criteria were as follows: acute coronary syndrome (ACS), known old myocardial infarction (OMI), frequent premature beat, atrial fibrillation, contraindications for iodinated contrast agent, serious coronary calcification and motion artifacts affecting measures of stenoses, and patient with revascularization (including percutaneous coronary intervention or bypass graft). All patients gave formal written consent approved by our Institution Ethics Committee.

### 2.2. MPI

#### 2.2.1. Imaging Acquisition

All patients underwent a 2-day stress/rest electrocardiography-gated MPI protocol. Exercise stress/rest gated MPIs were performed on a SPECT scanner (GE Millennium VG5 SPECT) before or after CTCA. Beta-blockers, calcium channel blockers, and nitrates were discontinued at least 24 h before MPI. The exercise stress tests were performed according to a modified Bruce's protocol on a bicycle ergometer with a 12-lead ECG, and blood pressure measurements were taken at the baseline and every 2 min during the whole procedure. The endpoints for the stress tests included any one of the following indexes: reaching target heartbeat ((220-age in years) × 85%), ischemic ST-segment horizontal or downslope depression of ≥2 mm, emergence of typical angina, severe cardiac arrhythmia, hypertension (≥240/120 mmHg), and a fall of systolic pressure ≥ 40 mmHg. At the peak of exercise, a 925-MBq dose of  ^99m^Tc-MIBI was injected into the bloodstream through the vein, and the patient continued to pedal for an additional 1 min. The ECG and blood pressure were monitored before and throughout the test and again after the injection. The acquisition for stress gated-SPECT study was performed about 1 h after injection. Rest studies started acquisition about 1.5 h after injection by using the same amount of doses. The acquisition parameters were listed as follows: a low-energy, high-resolution collimator; a 20% symmetric window at 140 keV; a 64 × 64 matrix; an elliptic orbit with step-and-shoot acquisition at 6° intervals over 180° from the right anterior oblique 45° to left posterior oblique 45°; 25 s dwell time per stop. Acquisitions were gated at 8 frames per R-R cycle with a 50% window of accepted heart rate.

#### 2.2.2. Image Reconstruction and Interpretation

All data were transferred to an eNTEGRA workstation and reconstructed using an iterative reconstruction algorithm (2 iterations and 10 subsets, Butterworth prefiltering function, a gradient order of 5.0, and a frequent cutoff of 0.25) without X-ray attenuation correction. Images were reconstructed into short axial, horizontal axial, and vertical long axial sections. At the same time, polar maps, wall motion, and wall thickening were obtained using a special software package (ECT toolbox and Multidim).

Semiquantitative visual judgment was made by consensus of two experienced observers aware of the size, weight, and gender of the patients but not the results of their CTCA or ICA assessments. The left ventricular myocardium was divided into 17 segments according to the American Heart Association [3]. Each segment was scored automatically by use of ECToolbox software based on a 5-point scoring system (0, normal uptake; 1, mild decreased uptake; 2, moderate decreased uptake; 3, severely decreased uptake; 4, absence of uptake). The sum of the stress scores of all segments (SSS) and the sum of the rest scores of all segments (SRS) were determined from these values. A summed difference score (SDS) was calculated as the difference between SSS and SRS. SDS > 1 was defined as reversible perfusion defect indicating ischemia. The results of MPIs were divided into two categories: negative MPI, defined as having homogenous radioactive distributions in myocardium and no defective segments noticed for both stress and rest scans; positive MPI, including reversible and fixed perfusion defects. Reversible pattern of localized segments of decreased perfusion at stress were no longer seen or demonstrated partial improvement at rest. Fixed pattern showed that localized segments of decreased perfusion were unchanged between the stress and rest images. Reversible perfusion defects were considered to represent myocardial ischemia. Fixed perfusion defects with concurrent regional wall motion abnormalities were considered to be myocardial scar.

### 2.3. CTCA

#### 2.3.1. Image Acquisition

All scans were performed on a 64-slice CT scanner (GE Light Speed VCT). Patients were required to fast 4 h before CT imaging. Patients with pre-scan heart rates above 65 b.p.m. needed to take a beta-blocker (Betaloc tablets, 25–100 mg). Before starting scans, patients were required to take 0.5 mg nitroglycerin sublingually. The acquisition parameters included 350 ms rotation time, 120 KV tube voltage, 650–800 mA tube current, and 0.6 mm collimation. Scan scope range was between the carina of the trachea and 2 cm below the diaphragm. The total scan time was about 12 s. In brief, a bolus of 20 mL contrast agent iohexol (350 mg/mL) was delivered intravenously through an antecubital vein at a flow rate of 4 mL/s using high-pressure injector for prescan. Bolus tracking technique was performed with a region of interest placed in the ascending aorta to record the peak time of enhancement and confirm the delay time of starting acquisition. A bolus of 70–80 mL of iohexol was then intravenously injected into the bloodstream at a speed of 4-5 mL/s through an antecubital vein. At the same time, the patients were required to hold their breath throughout the scanning procedure.

#### 2.3.2. Image Reconstruction and Interpretation

The acquired data were transferred to a GE Advanced Workstation, and a late diastole phase (75% R-R time) was automatically reconstructed. If the qualities of reconstructed images were not satisfactory, the images of 45%–85% R-R time were reconstructed and reviewed to select the best reconstructed images which clearly depicted coronary arteries. Finally, the images were displayed on screen in the form of multiple planar reformations (MPRs), virtual rendering volume (VR), and maximal intensity projection (MIP). The images were visually and independently evaluated by two experienced readers (with more than three years of experience in cardiovascular imaging) who were both blinded to the MPI findings. Upon reaching a consensus, the two readers agreed on the final diagnosis. Coronary arteries were subdivided according to a 15-segment model proposed by the American Heart Association [4]. Each segment was visually assessed and reported as ≥50% or <50% and allocated to the left main (LM), left anterior descending (LAD), left circumflex (LCX), and right coronary arteries (RCAs). The narrowest lesion was regarded as a final diagnosis for diffuse or multiple stenoses in a single vessel. Visual assessment of vessel narrowing of 50% or greater for at least one main vessel was defined as significant stenosis (positive CTCA), otherwise negative CTCA.

### 2.4. ICA

Conventional ICA was according to the standard Judkins catheter technique. Multiple views for each coronary artery were obtained digitally and stored in a designated workstation after intracoronary application of iodinated contrast agent. The angiograms were observed and evaluated by two experienced interventional cardiologists who were blinded to the CTCA and MPI findings. The readers agreed on the final diagnosis upon reaching a consensus. Visual assessment of vessel narrowing of 50% or greater for at least one main vessel was defined as significant stenosis (positive ICA), otherwise negative ICA.

## 3. Data Analysis and Statistics

The percentages of positive or negative CTCA among patients with positive or negative MPI results were calculated, and the percentages of positive or negative MPI among patients with positive or negative CTCA results were also calculated.

Abnormal perfusion area (defect) was allocated to coronary artery territories according to the reference as described previously [5]. Defects in the anterior wall and septal wall were allocated to the left anterior descending coronary artery (LAD); defects in the lateral wall, to the right coronary artery (RCA). Defects affecting both LAD region and LCX region were considered as left main artery (LM) lesion. In the watershed regions, allocation was determined according to the main extension of the defect onto the lateral, anterior, or inferior wall. The number of cases with vessel narrowing of ≥50% on CTCA with corresponding perfusion defect on MPI was calculated for positive predictive value of CTCA, and the number of cases with vessel narrowing of <50% without corresponding perfusion defect was also calculated for negative predictive value of CTCA.

Using ICA as referenced standard, diagnostic sensitivity, specificity, and accuracy of MPI or CTCA for CAD were calculated and statistically compared, respectively. Mean values were compared using the double-tailed *t*-test, and comparison of statistical differences between enumeration data was performed using Chi-square test. A *P* value of <0.05 was considered significant. Data were analyzed using STATA software version 13.0.

## 4. Results

The clinical characteristics of patients for different groups are shown in Table 1.

### 4.1. Relation between MPI and CTCA

The results for CTCA with corresponding MPI were showed in Table 2. Using MPI results as referred criteria, the positive predictive value for positive CTCA predicting abnormal haemodynamics is low (40.2%), but its negative predictive value for eliminating perfusion abnormalities is relatively high (94.4%), and the difference between them has statistic significance (*χ* ^2^ = 90.3, *P* < 0.01).

**Figure 1:**
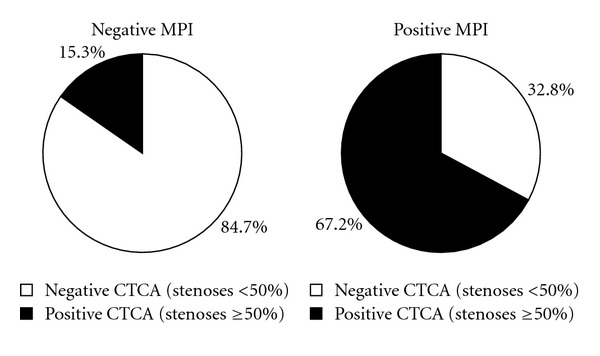
The percentages of negative or positive CTCA among patients with negative or positive MPI results.

**Figure 2:**
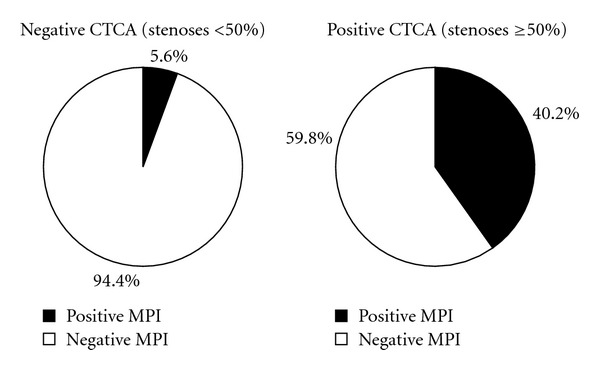
The percentages of negative or positive MPI among patients with negative or positive CTCA results.

Among patients with negative MPI results, negative or positive CTCA occupied 84.7% or 15.3%, respectively. Among patients with positive MPI results, positive or negative CTCA occupied 67.2% or 32.8%, respectively. Among patients with negative CTCA results, negative or positive MPI occupied 94.4% or 5.6%, respectively; among patients with positive CTCA results, positive or negative MPI occupied 40.2% or 59.8%, respectively. The above results were represented on Figures 1 and 2 in the form of pie charts.

### 4.2. Comparison of MPI and CTCA to Conventional ICA in 56 Patients

With ICA results regarded as reference, the performance indexes of MPI and CTCA detecting CAD were listed in Table 3. The data showed that both MPI and CTCA had good sensitivity, specificity, and accuracy for the detection of obstructive CAD compared with ICA (*P* > 0.05).

**Figure 3:**
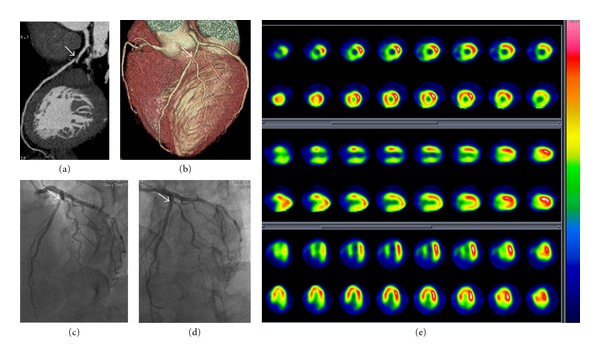
Patient with stenosis in the proximal segment of LAD classified as ≥50% on CTCA related with large and reversible perfusion defects (ischemia) in apex, anterior, and septal wall on MPI. (a) and (b) Multiple reformation (MPR) and maximal intensity projection (MIP) on CTCA showed noncalcification plaque in the proximal segment of LAD adjacent to the ostia of diagonal branch causing a significant stenosis (arrows), and no positive results on other main arteries were found. (c) ICA of left coronary arteries showed severe stenosis (arrow) in the same segment of LAD compared with CTCA. (d) ICA of left coronary arteries showed successful stenting (arrow) for the lesion in the proximal segment of LAD. (e) SPECT MPI study at stress (odd row) and rest (even row) showed large and reversible perfusion defects in the area of apex, anterior and septal wall corresponding to the territory of LAD, indicating large and severe myocardial ischemia.

It was important to note that one patient with balanced three-vessel stenoses (narrowing of 80% for LAD, LCX, and RCA) was missed by MPI among seven negative patients who underwent SPECT compared with ICA.  Figure 3 presents a typical case of a 55-year-old man with intermittent angina pectoris for half a year. Anatomic and physiologic information provided by CTCA and MPI showed that interventional therapy should be directed to the lesion in the proximal segment of LAD. The patient finally received stent implantation for the lesion located in the LAD with success. A typical case is shown in Figure 3.

## 5. Discussion

The diagnostic principle of CAD is very different for MPI and CTCA, and MPI has abilities to show the extent and severity of ischemia by visual or semiquantitative method, but it cannot show the morphology and plaques of coronary arteries. CTCA can directly depict the site and severity of coronary lesions. In addition, the length of stenosis, distribution, magnitude, and even composition of plaque can be precisely revealed and classified (calcified versus noncalcified) [6, 7]. But CTCA does not directly provide the hemodynamic significance related to the abnormalities of coronary arteries, which is very important in developing the therapeutic strategies for CAD. The coronal stenoses found on CTCA do not always lead to decreased perfusion to the myocardium. Previous study [8] showed that the severity of coronary stenosis was only a moderate surrogate among factors affecting myocardial blood perfusion, not sole decisive factor. Other factors affecting myocardial perfusion include length and shape of narrowing, eccentricity of plaque, and serial stenosis. Salm et al. [9] reported that MPI was normal in 50% of angiographically significant lesions. Particularly, lesions with an intermediate stenosis severity may have a great variability in hemodynamic significance [10].

In this study, negative MPI occupying 59.8% among patients with positive CTCA indicated that more than 50% ratio among patients with obvious stenoses (≥50%) on CTCA did not have hemodynamic significance of myocardial perfusion. Our results are consistent with the finding reported by Schuijf et al. [11], and it showed that only 45% of patients with an abnormal MSCT had abnormal MPI, even in patients with obstructive CAD on MSCT, 50% still had a normal MPI. Just as what is mentioned in Schuijf's study, myocardial perfusion imaging and MSCT provide different and complementary information on CAD, namely, detection of atherosclerosis versus detection of ischemia. Coronary stenosis itself usually does not sufficiently predict corresponding hemodynamic abnormality. Whether coronary stenosis causes corresponding abnormal hemodynamics or not possesses considerable variations, especially for moderate stenoses (narrowing between 40% and 70%).

As the results found in this study, it clearly shows that CTCA has higher negative predictive value for eliminating perfusion defects. Hausleiter et al. [12] had proposed that coronary multislice CT could be a suitable means for the management of patients with an intermediate pretest likelihood of significant CAD. Invasive angiography would not be necessary if coronary MSCT angiography demonstrates normal coronary arteries. But the positive predictive value for CTCA reflecting haemodynamics was revealed to be relative low from this study. Therefore, functional imaging still needs to be done to reveal pathophysiologic changes related to coronary lesions. With coronary stenosis worsening, the severity of stenosis could be one of the most critical factors affecting corresponding myocardial perfusion [9, 13].

It should be kept in mind that negative MPI does not exclude coronary atherosclerosis and/or coronal stenoses, even existence of obvious stenoses, but patients with negative MPI results are usually thought to have a low risk of hard cardiac events. Patients with angiographically confirmed CAD, but negative MPI results have been shown to have similar low event rates [14, 15]. CTCA provides with very different information on CAD compared with MPI, which has abilities to detect plaque burden of coronary atherosclerosis and severity of stenoses at early stage. With plaque burden and stenosis worsen, the prevalence of corresponding abnormal hemodynamics is increasing, but coronal plaque burden and stenosis are not synonyms for myocardial ischemia.

CTCA not only has the abilities to diagnose stenoses of coronary artery, but also shows the prognostic value for CAD. The study from van Werkhoven et al. [16] indicated that MSCT is an independent predictor of events and provides incremental prognostic value to MPI, combined anatomical and functional assessment may allow improved risk stratification. In addition, early identification of CAD with CTCA may also be useful for risk stratification. Non-invasive anatomic assessment of plaque burden, location, composition, and remodeling using CTCA may provide prognostically relevant information. This information has been shown to be incremental to the Framingham risk score, coronary artery calcium scoring, and myocardial perfusion imaging [17].

Therefore, MPI and CTCA play a complementary rather than excluding role in diagnosing and evaluating CAD. According to guidelines of American cardiac intervention therapy and radionuclide imaging, noninvasive morphological and functional method should be used before performing revascularization on patients with chronic stable angina, and coronary stenosis with corresponding ischemia is one of the major indications for performing revascularization, while conservative therapy should be adopted when there are no significant stenoses or corresponding ischemia [18–20]. So the incremental values for guiding therapeutic strategies using combined CTCA and MPI for the detection of CAD are summarized as follows: first, a patient with neither significant coronary stenoses nor perfusion defects should undergo primary prevention and control of risk factors; second, a patient with significant coronary stenoses and no corresponding perfusion defects should undergo aggressive medical therapy and control of risk factors; third, a patient with significant coronary stenoses and corresponding perfusion defects should undergo active revascularization to obtain better prognosis rather than conservative therapy [21, 22]. CTCA and MPI are helpful in making the correct decision to insure patients benefit from treatments when noninvasively obtaining functional and anatomic information on coronary lesions.

It should be aware of the diagnostic principle of MPI being normal or abnormal depending on the relative distribution of radiopharmaceuticals in myocardium, so MPI possibly just detects abnormal territories corresponding to one or two relatively severe stenoses of coronary arteries for patients with three-vessel disease, and MPI even is negative for balanced reduction of perfusion to the myocardium for patients with balanced three-vessel stenoses [23, 24]. Therefore, MPI may miss some patients with balanced three-vessel disease, whereas CTCA is unlikely to miss severe or extensive coronary atherosclerosis [25]. For coronal microvascular disease causing ischemia, such as X syndrome, no matter CTCA or ICA cannot make a direct diagnosis because of limitation of spatial resolution, while MPI can make a diagnosis of myocardial ischemia for X syndrome by detecting ischemia [26, 27].

Although the rate of positive MPI was less than 50% among patients with significant stenoses on CTCA in this study, there was no statistically difference for the indexes of diagnosing CAD between MPI and CTCA in fifty six patients who underwent ICA in the same period. The reason may be related to the different morbidity of CAD between total patients and the patients who underwent ICA. The morbidity and stenotic severity in patients who underwent ICA were probably increased compared with total patients in this study for the reason of primarily being screened by clinicians. As has been stated, stenoses maybe one of main factors affecting corresponding hemodynamics with the severity of coronal stenoses worsen. So the diagnostic performances between MPI and CTCA tend to reach an agreement for patients with higher morbidity and severity of CAD.

In conclusion, both MPI and CTCA have good diagnostic performance for CAD, and they provide different and complementary information for diagnosis and evaluation of CAD, namely, detection of ischemia versus detection of atherosclerosis, which are quite different but have a definite internal link for each other.

## Figures and Tables

**Table 1:** Characteristics of the population for positive and negative groups of MPI and CTCA.

Parameters	Positive MPI	Negative MPI	*P* value	Positive CTCA	Negative CTCA	*P* value
	(*n* = 67)	(*n* = 437)		(*n* = 112)	(*n* = 392)	
Age	54.0 ± 10.8	51.8 ± 11.8	*P* > 0.05	49.8 ± 11.8	50.6 ± 11.9	*P* < 0.05
Prior myocardial infarction	None	None	—	None	None	—
Prior coronary revascularization	None	None	—	None	None	—
Risk factors for CAD						
Diabetes mellitus	19	10	*P* < 0.05	18	11	*P* < 0.05
Hypertension	55	32	*P* < 0.05	60	27	*P* < 0.05
Hypercholesterolemia	34	213	*P* > 0.05	89	158	*P* < 0.05
Family history of CAD	42	56	*P* < 0.05	51	47	*P* < 0.05
Smoking history	23	88	*P* < 0.05	35	76	*P* < 0.05

**Table 2:** The relations between MPI and CTCA results.

CTCA	MPI		Total
	Positive	Negative
Positive	45	67	112
Negative	22	370	392

Total	67	437	504

**Table 3:** The diagnostic indexes of MPI and CTCA and comparison between them in 56 patients.

Indexes	MPI	CTCA	*P* values
True positive	24	27	
True negative	20	22	
False positive	5	3	
False negative	7	4	
Sensitivity	82.8%	90.0%	0.67^∗^ (0.19)
Specificity	74.1%	84.6%	0.34^∗^ (0.90)
Accuracy	78.6%	87.5%	0.21^∗^ (1.59)

^∗^Chi-square test, value of *χ* ^2^ in brackets.
